# Medical Journal, Readers, Success: An Inseparable Trinity

**DOI:** 10.3390/jcm13030856

**Published:** 2024-02-01

**Authors:** Emmanuel Andrès

**Affiliations:** Department of Internal Medicine, University Hospital of Strasbourg, 67000 Strasbourg, France; emmanuel.andres@chru-strasbourg.fr

At the start of this new year, the Editorial Board of the *Journal of Clinical Medicine* (*J. Clin. Med.*) would like to send you its most sincere wishes for health, happiness and success. May this New Year 2024 be rich in discoveries, scientific advances and rewarding moments in your professional and personal careers.

May every article in *J. Clin. Med.* that you read in this new year enrich your knowledge and inspire you in the care of your patients. May your commitment to the medical field continue to bring lasting benefits to the lives of those who place their trust in you.

In these uncertain times, when the world and society are changing, medicine is becoming more personal, and both individuals and the field of medicine must reinvent themselves [1]. May 2024 be a year when your projects come to fruition, when collaboration between colleagues and medical research bear fruit, and when the passion that drives you makes everyday life less gloomy and the future brighter.

*J. Clin. Med.* is one of the reference journals for general medical journals, including *The New England Journal of Medicine*, *The Lancet*, *The British Medical Journal* and *The Annals of Internal Medicine*. Over the last 5 years, its impact factor has fluctuated between 3.9 and 5.6 [2,3]. This is one of the hallmarks of the journal’s excellence, as are its significant citation indices [3]. The year of 2023 came to an end, and in the past year, *J. Clin Med.* received an Impact Factor of 3.9 (Medicine, General & Internal, Q2 ranking 58/167), and a Cite Score of 5.4 (General Medicine, ranking 137/830). In the new year, we are continuing to strive for the advancement of science and for publishing higher quality research and reviews.

Among the most cited articles in *J. Clin. Med.* in the last 2–3 years on topical subjects, such as COVID-19 or the role of the microbiota, we would like to highlight the work of:

- Riad A. et al. on the prevalence of COVID-19 vaccine side effects among healthcare workers in the Czech Republic [4];
- Fernández-de-las-Peñas C. et al. on female sex is a risk factor associated with long-term post-COVID-related-symptoms but not with COVID-19 symptoms: The LONG-COVID-EXP-CM Multicenter Study [5];
- Sarkar et al. on association between early-life gut microbiota and long-term health and diseases [6].

To meet this ambition of playing in the court of the great journals, *J. Clin. Med.* relies on a clear and transparent editorial policy, outlining selection criteria, review processes and ethical standards; high standards of scientific rigor in the selection, review and publication of articles; a rigorous and impartial peer review process; and financial independence from industrial players in the healthcare world [3].

The accessibility of the content in *J. Clin. Med.*, via an open access policy [3], enables knowledge to be disseminated more widely and enhances the journal’s impact on the global medical community.

Reader participation is a dynamic and essential aspect of the life of a medical journal, contributing to its evolution, its scientific excellence and its impact on the medical community. With this in mind, the Editorial Board of *J. Clin. Med*. is counting on you more than ever.

You can submit original articles, literature reviews, case studies or other relevant contributions. This enriches the journal’s content and offers a diversity of perspectives.

Readers can share their feedback on published articles. This can include constructive comments, suggestions for improvement or questions for the authors, thus contributing to a more in-depth scientific discussion.

You can suggest themes, topics or areas of research that you would like to see addressed in the magazine. This can help guide editorial development in line with the interests of the community, and ensure that the journal meets your expectations. In this context, the MDPI Editor of *J. Clin. Med.* offers discussion forums, webinars and other interactive platforms [7].

Readers play a key role in the success of a journal. They must participate, make their voices heard, bring the journal to life and help it evolve.

We thank you for your loyalty to our medical journal, and are honored to be able to support your ongoing quest for knowledge and excellence. May this year be for you a wonderful adventure of success, fulfillment and sharing. Happy New Year 2024!
